# Pathogenesis of Aleutian Mink Disease Virus Infection—Comparison of Natural Transmission with Experimental Aerosol and Intraperitoneal Inoculation

**DOI:** 10.3390/pathogens15050494

**Published:** 2026-05-03

**Authors:** Mette Sif Hansen, Mariann Chriél, Lars Erik Larsen, Charlotte Kristiane Hjulsager

**Affiliations:** National Veterinary Institute, Technical University of Denmark, 2800 Kongens Lyngby, Denmark; mariann.chriel@gmail.com (M.C.); lael@sund.ku.dk (L.E.L.); ckhj@ssi.dk (C.K.H.)

**Keywords:** aleutian disease (AD), mink plasmacytosis, *Neogale vison*, aerosol inoculation, sentinel, transmission

## Abstract

Though intraperitoneal (IP) inoculation is not the natural pathway of Aleutian Mink Disease Virus (AMDV) infection in mink, it is frequently used experimentally. To investigate AMDV pathogenesis, we compared the effects of IP, aerosol (AE), and natural infection in mink. Forty-six sapphire mink were divided into groups: negative controls, IP and AE AMDV-inoculated mink, and sentinels exposed to IP-inoculated mink for two-week periods. Mink in the control, IP, and AE groups were euthanized 2, 5, or 10 weeks post-inoculation. The mink were tested for AMDV antibodies and by PCR on serum samples throughout the study, and by PCR and histology in organs after euthanasia. The sentinel mink were introduced to determine when the risk of natural transmission was highest. AMDV was detected in the sentinels exposed during weeks 3–6, indicating that AMDV transmission risk is highest early in infection, before antibody-positive animals can be detected on the farm. Infection in the AE group progressed more gradually than in the IP group, which developed more pronounced lesions and higher viral loads in the liver. Compared to IP inoculation, the aerosol model provides a superior experimental approach for studying natural infection and transmission of AMDV.

## 1. Introduction

Aleutian Mink Disease Virus (AMDV) is a parvovirus infection in mink (*Neogale vison* [*Neovison vison*]) that causes economic and animal welfare challenges in fur-producing countries. AMDV is a DNA virus, belonging to the genus *Amdoparvovirus* within the *Parvoviridae* family [1], and several strains of AMDV exist [2,3,4]. The disease in adult mink is referred to as Aleutian disease (AD) and mink plasmacytosis, based on the inflammation characterized by multiorgan infiltration of mononuclear cells, including many plasma cells [5,6,7]. Histopathologically, AD causes characteristic tissue changes; however, the final diagnosis must be verified by detection of AMDV DNA in blood or tissues by PCR analysis or presence of AMDV antibodies in blood samples [8]. The virus is stable and can persist for a long time in the environment, and it is, therefore, extremely difficult to decontaminate infected mink farms [9,10].

AMDV can be transmitted vertically and horizontally. The virus is present in saliva, feces, urine, blood, and multiple organs and can be transmitted by fomites. The natural route of infection is believed to be by oronasal uptake of the virus through the feed or by inhalation of aerosols [6,11].

The outcome of infection depends upon several parameters, such as age and genotype of the host, with Aleutian mink being more susceptible to developing severe AD; viral load and AMDV strain also affect disease development [12,13]. Since no effective treatments or prophylaxis exist, the usual control strategy on infected farms is stamping out of AMDV antibody-positive animals; however, this approach has proven to be inefficient [2,10,14]. When initiating eradication strategies, detailed knowledge of virus transmission and pathogenesis under natural conditions is important, parameters that remain insufficiently understood in AD.

The experimental challenge of mink is often used to study the AMDV infection, aiming at developing vaccines, therapy, and control strategies. In these studies, the most frequently used inoculation route is intraperitoneal (IP) injection of organ homogenate from AMDV-infected mink, since pathogenic strains of AMDV are not culturable in vitro [15]. Even though IP inoculation is not the natural route of infection, this inoculation route is used to ensure that identical infection doses are administered and that the infection is established, since oral or intranasal inoculation is less efficient in causing infection, and the viral dose might differ between animals [5,16]. From other animal species and diseases, it is known that the route of inoculation is important for disease development when examining infections with viruses, bacteria, and parasites [17,18,19,20]. Similarly, a study showed a tendency for mink inoculated IP with AMDV to first develop lesions in the liver, followed by later involvement of other organs [5]. This was believed to be due to the IP route of inoculation favoring the liver, as this organ is in the abdominal cavity and has a large surface area and high blood flow. In contrast, it is expected that after IP inoculation, AMDV does not reach, e.g., the lungs and brain, which are in the thorax and cranium, respectively, until viremia has occurred.

The aim of the study was to examine the effect of various inoculation pathways on the pathogenesis of AD. Development of disease and pathology in mink infected by the “classical” experimental IP inoculation route was compared with mink exposed to the virus by intranasal aerosol inoculation (AE) and by sentinel contact with the IP-infected mink. The sentinels were introduced at different times to study at which stage of the infection, the risk of becoming infected by horizontal transmission was highest.

## 2. Materials and Methods

### 2.1. Ethical Statement

The present study describes an animal experiment that was carried out in accordance with the experimental protocol approved on 24 November 2016 by the Danish Animal Experiments Inspectorate (license no. 2016-15-0201-01106).

### 2.2. Mink

Forty-six sapphire female (*n* = 21) and male (*n* = 25) mink kits (*Neogale vison*) between 15 and 16 weeks of age were purchased from a commercial AMDV-free mink farm on Zealand, Denmark, and placed at the experimental high-containment test facility at the National Veterinary Institute, Technical University of Denmark (DTU-Vet), Lindholm, Denmark. The farm of origin did not vaccinate and had no records of previous AMDV, mink enteritis virus, morbillivirus, influenza, or other serious infections. Before in-housing, all mink were tested and found negative for antibodies to AMDV using counter-current immunoelectrophoresis [21].

Sapphire mink were used for the study, since they carry the Aleutian gene, making them more susceptible to AD [13].

### 2.3. Experimental Design

**Figure 1:**
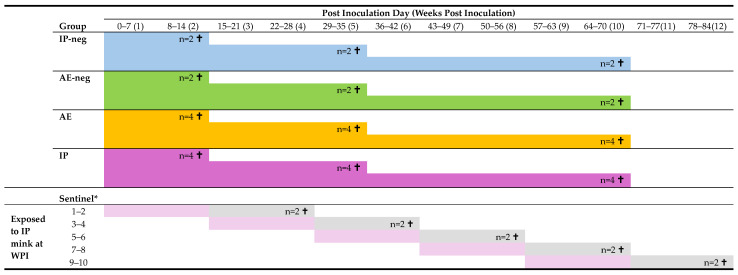
Experimental design of the Aleutian Mink Disease Virus (AMDV) pathogenesis study. Mink were inoculated by aerosol or intraperitoneal inoculation with mock (group AE-neg and IP-neg) or AMDV (group AE and IP), and euthanized 2, 5, or 10 weeks post-inoculation (WPI). n = number of mink euthanized. † Mink were euthanized on the last day of the period. * Sentinel mink were housed together with the IP-inoculated mink for two weeks (light purple) and then transferred to a room without other mink for two weeks (light grey) before euthanasia.

The mink were housed indoors in five isolation rooms designed for animal experiments with 12 standard mink cages, each equipped with a straw-filled wooden nest box. The mink were divided into 5 groups (Figure 1): six negative control mink, inoculated intraperitoneally with mock (IP-neg); six negative control mink, aerosol-inoculated with mock (AE-neg); 12 mink inoculated intraperitoneally with AMDV (IP); 12 mink aerosol-inoculated with AMDV (AE); and ten sentinel mink. The small group sizes were intended to examine tendencies of infection dynamics and could not support definitive statistical analyses.

The groups were divided into separate isolation rooms with group IP-neg and AE-neg housed together, group AE was housed alone, and group IP was housed with two sentinels (one male and one female) that were replaced with two new sentinels after two weeks (see below). In the IP-neg and AE-neg groups, one male and one female shared a cage. Sentinels and mink in the AE and IP groups were housed individually. In the AE group, solid partitions were installed between the cages. Daily, the mink were fed standard commercial mink feed. Upon arrival, all mink were found healthy after clinical assessment and acclimatized for six days before inoculation at post-inoculation day (PID) 0. All mink were observed daily and clinically assessed based on the scoring system developed by Jensen et al. (2016) [5]. Recorded parameters were general condition, respiration, appetite, and feces. Mink showing severe or prolonged clinical signs during the trial period were euthanized and frozen at −20 °C until necropsy. The evaluated welfare parameters were weight loss (loss of 10% bodyweight compared to the weight at the previous weighing), lack of appetite, reduced well-being, gastrointestinal disorders, respiratory distress, urinary tract symptoms, or wounds. Each week, the mink were weighed, and a blood sample was obtained from the cephalic vein in the foreleg under manual restraint. At the end of the experiment, the mink were sedated by intramuscular (IM) injection of ketamine (10–15 mg/kg) and xylazine (0.5–2 mg/kg), and euthanized by an intracardial injection of pentobarbital (140 mg/kg).

Two mink from the IP-neg and AE-neg groups, and four mink from the AE and IP groups were euthanized after 2 weeks, 5 weeks, and 10 weeks post-inoculation (WPI), respectively. The mink were necropsied, and tissues were sampled for histology and PCR analysis (see below).

### 2.4. Experimental Inoculation with AMDV or Mock

**Figure 2:**
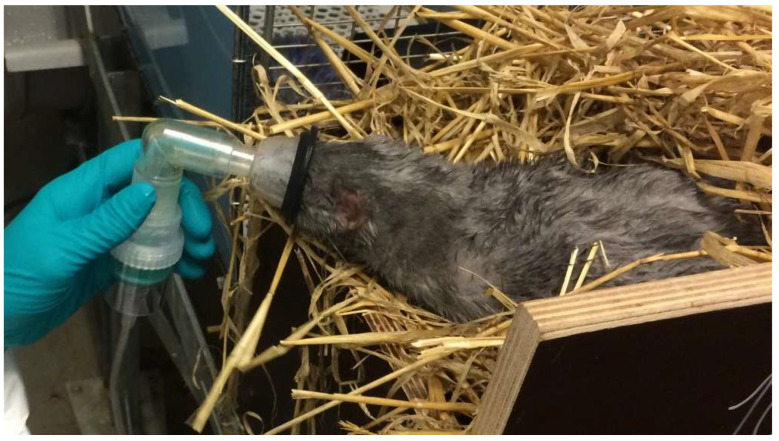
Aerosol inoculation of anesthetized mink was performed with a nebulizer chamber connected to an anesthesia mask covering the nose and mouth of the mink.

The virus inoculum was prepared from spleen homogenate supernatants from mink experimentally infected with the Danish AMDV Saeby field strain “AMDV/Denmark/Saeby/52-1/2016” by dilution 1:10,000 in PBS (8.2 g/L NaCl; 2.8 g/L Na_2_HPO_4_; 0.3 g/L KH_2_PO_4_; pH 7.1) with 4% penicillin-streptomycin to obtain a virus titer of 6.97 × 10^10^ copies/mL quantified by qPCR. The diluted homogenate was stored at –80 °C until use. Before inoculation, the mink were anesthetized with an IM injection of ketamine (10–15 mg/kg) and xylazine (0.5–2 mg/kg). For IP inoculation, 1 mL of mock (PBS with 4% penicillin-neomycin (IP-neg group)) or 1 mL of AMDV spleen homogenate (IP group) was injected into the abdominal cavity. AE inoculation was performed using a nebulizer chamber (Hudson Micro Mist, Mediq Denmark A/S, Broendby, Denmark) connected to an anesthesia mask (Jørgen Kruuse A/S, Langeskov, Denmark) covering the nose and mouth of the mink (Figure 2). The nebulizer allowed passage of particle sizes up to 2.1 μm. The nebulizer chamber was filled with 2 mL of mock (AE-neg group) or 2 mL of AMDV spleen homogenate (AE group), after which the mink inhaled for 2 min. If the volume of the residual inoculum exceeded 1.1 mL, the mink inhaled for another minute in the mask. The inhaled volumes of mink euthanized at 2 WPI were: mock-inoculated 1.4 and 1.5 mL; AMDV-inoculated 1.4, 1.4, 1.6, and 1.7 mL. In mink euthanized at 5 WPI: mock-inoculated 1.2 and 1.7 mL; AMDV-inoculated 0.9, 1.2, 1.2, and 1.6 mL. In mink euthanized at 10 WPI: mock-inoculated 1.3 and 1.4 mL; AMDV-inoculated 0.7, 0.8, 1.0, and 1.4 mL. Uptake was confirmed by observing aerosols delivered into the anesthesia mask, steady respiration of the mink, and the absence of fluid (inoculum) dripping from the connecting tubes, mask, or the nose/mouth of the mink after the inoculation procedure.

### 2.5. Sentinels

Ten mink served as sentinels, i.e., animals that were not inoculated, but were exposed to natural infection from the IP-inoculated mink, by direct and indirect contact (aerosols, feces). At the start of the experiment, all ten sentinel mink were housed together with the negative control animals. Every 2 weeks (at PID 0, 14, 28, 42, 56), two sentinels were placed individually in empty, clean cages in the room with the IP-inoculated mink (Figure 1). The cages were positioned so each sentinel had at least one AMDV-inoculated mink on each side, allowing direct nose-to-nose contact and exposure to aerosols and feces. After 2 weeks of co-housing, the two sentinels were transferred to a clean cage in a room without other mink and kept for an additional 2 weeks until euthanasia. The sentinel cadavers were frozen at −20 °C until necropsy. The same procedure was repeated four times. Thus, the groups of sentinels were exposed to the IP-inoculated mink at WPI 1–2, 3–4, 5–6, 7–8, and 9–10, respectively. The sentinels were blood sampled two days prior to exposure to the IP AMDV-inoculated mink, at euthanasia, and two to four times in the intermediate period.

### 2.6. Pathology

At necropsy, pathological findings were recorded. For histology, tissue samples from lung, spleen, liver, small intestine, mesenteric lymph node, kidney, and brain from all mink were fixed in 10% neutral buffered formalin, processed by routine methods, paraffin-embedded, cut in 3–5 µm sections, mounted on standard glass slides, and stained with hematoxylin and eosin.

By histology, organs were examined for the following changes indicative of AD [5,22]: perivascular infiltration of lymphocytes, plasma cells and macrophages (i.e., mononuclear cells) in all organs; infiltration of mononuclear cells in the intestinal lamina propria, in the liver parenchyma and portal triads, in the renal interstitial tissue, in the brain parenchyma and in the leptomeninges; and peribronchially in the lung. The inflammation in the lung, liver, intestine, kidney and brain was scored semiquantitatively, slightly modified after Jensen et al. (2016) [5] and given a numerical score of 0–4 depending on the degree of infiltration of mononuclear cells, as: none (no infiltration; 0), focal (small focal infiltration; 1), mild (few and small infiltrations; 2), moderate (several small or few disseminated infiltrations; 3) or massive (multifocal disseminated infiltrations; 4). Other findings not related to AMDV infection were also noted. The histologic scoring was performed blinded and in duplicate by an experienced pathologist. The final histopathological score was an average of the two scorings. Intra-observer agreement was examined using weighted kappa using GraphPad (https://www.graphpad.com/quickcalcs/kappa1/, accessed on 9 March 2026).

### 2.7. DNA Extraction and Quantitative PCR (qPCR) Analysis

Serum and tissues from lung, spleen, liver, small intestine, mesenteric lymph node, kidney, and brain from all mink were sampled and stored at −80 °C until use. QIAamp DNA Mini Kit (QIAGEN, Copenhagen, Denmark) was used to purify DNA from 200 µL of serum or 200 µL tissue homogenate in ATL Buffer (QIAGEN, Copenhagen, Denmark) as previously described [2], with the modification that DNA extraction was performed manually. AMDV quantitative real-time PCR (qPCR) was performed in a total volume of 25 µL, containing 3 µL template and a final concentration of 1x JumpStart *Taq* ReadyMix (JumpStart^TM^ *Taq* ReadyMix^TM^ For Quantitative PCR, Sigma-Aldrich, Søborg, Denmark), 300 nM of each primer (ADVNS-6F, 5′-CACTGGAAAAACCTTGCTAGC-3′ and ADVNS-6R 5′-CAAGGTTACCACACTCTT-3′), 250 nM hydrolysis probe ADV-NS1-6P (5′-FAM-CCAAACTTTCCATGGACTGACTGTGGCA-3′-BHQ1) and 3.5 mM MgCl_2_ (Sigma-Aldrich, Søborg, Denmark). Cycling was performed on a RotorGene Q PCR machine (QIAGEN, Copenhagen, Denmark) with autogain calibration performed before the first acquisition in the Green Channel at 60 °C and PCR cycling conditions: 94 °C, 2 min, followed by 40 cycles of 94 °C, 15 s; 60 °C, 60 s. Data were acquired in the Green Channel at the 60 °C step. A Cq threshold of 0.01 and an NTC threshold of 10% were used for data analysis. Quantification was performed against a standard curve consisting of 10-fold dilutions of a synthetic oligonucleotide representing the AMDV amplicon. An AMDV-G cell culture isolate was used as a positive purification control and PCR control. Nuclease-free water was used as a negative control for extraction and PCR.

### 2.8. Serology

Blood deposited on dried blood spot cards (DBS cards (Kopenhagen Fur, Glostrup, Denmark)) was examined for AMDV antibodies using an automated ELISA (ELISA Danad) at Kopenhagen Fur, Glostrup, Denmark [21]. The sensitivity and specificity of the ELISA were 0.8857 and 0.9984, respectively, and a cut-off value of 1.0 U was applied.

## 3. Results

### 3.1. Clinical Observations

**Figure 3:**
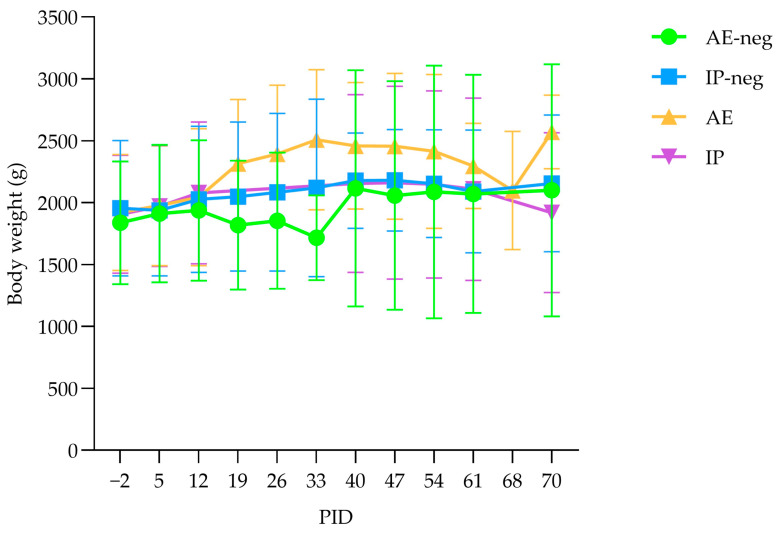
Development of mink body weights after aerosol (AE) or intraperitoneal (IP) inoculation with mock (group AE-neg (●) and IP-neg (■)) or Aleutian Mink Disease Virus (AMDV) (group IP (▼) and AE (▲)) at different post-inoculation days (PID). Presented as the mean body weight and the standard deviation of the number of mink in the groups at the specific time in the study.

During the experiment, transient periods of variable loss of appetite without affecting the general condition were observed in all groups, but most frequently among AMDV-inoculated mink. Other clinical observations included single-day episodes of reduced fecal consistency in a few mink across all groups. Two AE-inoculated mink were euthanized at PID 68, 2 days earlier than planned due to welfare issues. One mink was bleeding from the mouth, and the other was slightly depressed. There were no apparent differences between body weights among the mock and AMDV-inoculated mink (Figure 3).

### 3.2. Pathology

#### 3.2.1. Gross Pathology

All mink were in normal or good body condition. At necropsy 2 WPI, only a few animals had macroscopical lesions. Two mink from the AE group had a slightly enlarged spleen, and one from the IP group had decreased meningeal opacity. After 5 WPI, one of the AE mock-inoculated mink had consolidated lung tissue in the anterior lung lobe, and the other control mink had slight splenomegaly. In the AE-inoculated group, one mink had preputial urine discharge, another had an intracranial hematoma, and greenish gelatinous fecal soilage around the anus. In the AMDV IP-inoculated group, two mink had preputial urine discharge, and one had slight splenomegaly. At the end of the experiment (10 WPI), kidney lesions were present in three mink in the AE-inoculated group, represented by a granular surface of the kidney in two mink; one of these mink also had slight splenomegaly and enlarged caudal mesenteric lymph nodes, and the last mink had multiple renal petechiae and much tartar. This mink was euthanized at PID 68 due to oral hemorrhage. In the IP-inoculated group, one mink had an enlarged gall bladder, another had enlarged caudal mesenteric lymph nodes, and one had glomerulonephritis.

Among the sentinels, two mink, exposed to IP mink at WPI 1–2 and 3–4, respectively, had slight splenomegaly, and one (exposed to IP mink at 5–6 WPI) had a granular surface of the kidney.

#### 3.2.2. Histological Findings

**Figure 4:**
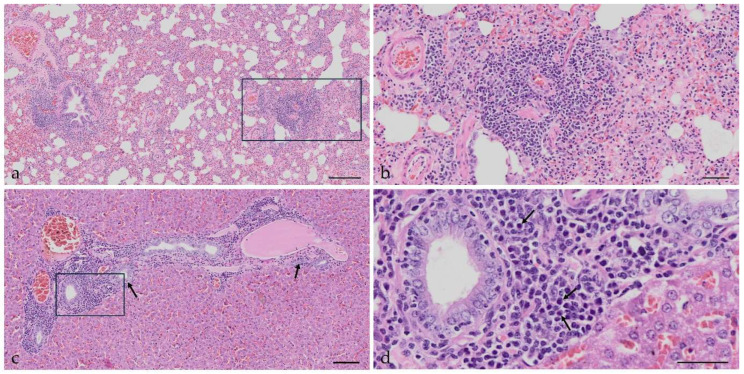
Histology from Aleutian Mink Disease Virus-infected mink euthanized 10 weeks post-inoculation, HE stain. (**a**) Lung from aerosol-inoculated mink with Aleutian Disease (AD) associated changes represented by massive peribronchiolar and perivascular mononuclear cell infiltration. Scalebar 200 μm. (**b**) Detail from a, showing massive perivascular mononuclear cell infiltration. Scalebar 50 μm. (**c**) Liver from intraperitoneal inoculated mink with AD-associated changes represented by massive infiltration of mononuclear cells and mild bile duct (arrows) hyperplasia in the portal triads. Scalebar 100 μm. (**d**) Detail from c, showing part of a portal triad with massive mononuclear cell infiltration consisting of many plasma cells (arrows), lymphocytes, and macrophages. Scalebar 50 μm.

The histological changes in the mock and AMDV-inoculated mink are presented in Table 1 and Figure 4. When comparing the two duplicate histological scorings, the weighted kappa showed an intra-observer agreement of 0.56 for the intestine, i.e., correlating with “moderate agreement”; 0.66 and 0.74 for lung and brain, respectively, interpreted as “substantial agreement”; and “almost perfect agreement” for liver and kidney, with kappa scores of 0.81 and 0.87, respectively.

Histological changes represented by mononuclear cell infiltrations in the lung were seen in all groups, both in mock and AMDV-inoculated mink. At WPI 2, three IP AMDV-inoculated mink had massive pulmonary changes, and one mink had moderate pulmonary changes, whereas the highest score among the AE AMDV-inoculated mink was three mink with mild changes. This pattern was reversed when looking at the negative control groups 2 WPI, where the pulmonary pathology was scored moderate or massive in the AE-neg group, and focal or mild in the IP-neg group. When comparing the remaining groups, the pulmonary changes were evenly distributed among focal, mild, moderate, and massive infiltrations, without a clear association with the inoculation route or infection status. Only among the AMDV-inoculated mink, there was a tendency to more pronounced inflammation at the end of the experiment (Table 1).

Apart from the lung, only focal infiltrations of mononuclear cells were seen in the liver, intestine, kidney, and brain of the IP-neg and AE-neg groups at 2, 5, and 10 WPI, although three IP mock-inoculated mink had mild infiltrations in the liver or in the intestine at 10 WPI.

Compared to the AE-inoculated mink, the IP-inoculated mink exhibited more pronounced liver lesions, mainly presented as moderate to massive mononuclear cell infiltrations, and the cellular infiltration increased with increasing duration of the infection. Bile duct hyperplasia was also observed (Table 1, Figure 4). Other findings in the liver were congestion and varying amounts of lipid accumulation in the hepatocytes, not related to any specific experimental groups.

There were only a few changes in the intestine after AE and IP inoculation, presented as focal or mild mononuclear cell infiltration; lesion development was non-related to inoculation route.

In the kidney, focal, mild, or moderate changes were seen at 5 WPI in both AMDV-inoculated groups. At 10 WPI, the cellular infiltration had increased, and the changes appeared more pronounced in the IP-inoculated group, as all mink in this group had massive mononuclear cell infiltrations, whereas this was only observed in half of the aerosol-inoculated mink. Other findings were mineral deposits in the tubules and lipid accumulation in the renal epithelium.

Brain lesions, mainly moderate cellular infiltrations, were present at 2 and 5 WPI, but only in the IP group. At 10 WPI, varying levels of brain lesions could be seen in all AMDV-inoculated mink.

The sentinels generally only showed minimal tissue changes. As in the other groups, the pulmonary lesions were frequent; thus, 8/10 sentinels had mild (*n* = 4) or moderate (*n* = 4) mononuclear cellular infiltration in the lung. Only the two sentinels exposed to IP mink at WPI 1–2 were without pulmonary changes; among the other sentinels, there was no correlation between the degree of pulmonary lesions and the weekly intervals in which they had been exposed. Two mink that were exposed to the IP-inoculated mink at WPI 9–10 and WPI 3–4, respectively, presented mild or moderate mononuclear cellular infiltration in the liver. The remaining sentinel mink only had focal changes in the liver, intestine, kidney, and brain.

### 3.3. Virus and Antibody Detection

All negative controls (IP-neg and AE-neg groups) remained negative for AMDV by qPCR in serum and all organs tested (Table 2 and Table 3, Tables S1 and S2) and did not develop AMDV antibodies (Table 4 and Table S2).

The AE and IP AMDV-inoculated mink necropsied at 2 WPI did not seroconvert before euthanasia (Table 4). At PID 26, antibodies were detected in 4/8 of the 5 and 10 WPI AE-inoculated mink, and at PID 54, all the remaining AE mink (*n* = 4) had seroconverted. Among the IP-inoculated mink, two mink seroconverted already at PID 19, and at PID 26, 8/8 mink were antibody-positive. Seroconversion was observed one to two weeks after AMDV was detected in the serum by PCR (Table 2 and Table 4).

In the AE-inoculated group, virus was not detected in serum or organs from the mink euthanized at 2 WPI (*n* = 4). In the 5 WPI group, the virus was detected in serum from 2/4 mink from PID 12 and in one further mink in this group from PID 33. Upon euthanasia, AMDV was detected in all organs tested. Among the mink euthanized, 10 WPI, 2/4 mink were shown to be viremic at PID 12, and by PID 40, all mink in this group had detectable levels of AMDV in the blood. Upon euthanasia, AMDV was detected in all organs. The level of virus in serum developed from low to high during the study period (Table 2), and viremic animals had high levels of virus in organs upon euthanasia, with no or minor differences between organs.

**Figure 5:**
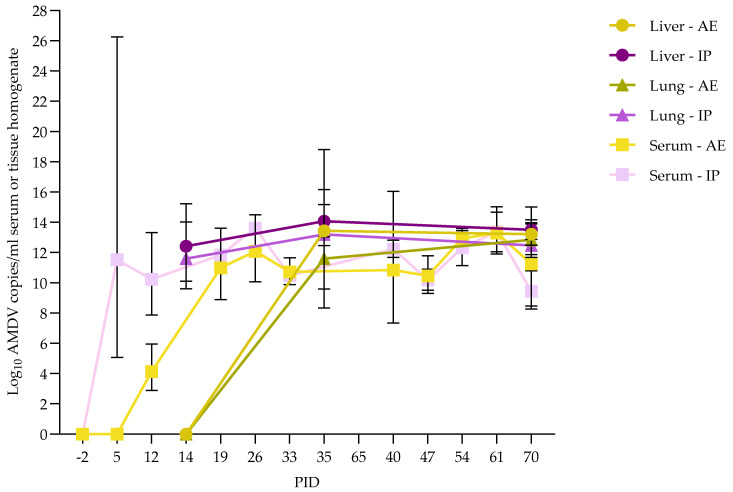
Development of viral load in liver, lung, and serum of mink inoculated with Aleutian Mink Disease Virus (AMDV) by aerosol (AE) or intraperitoneal (IP) route during the study. Averages of positive PCR results are presented as a geometrical mean with 95% confidence interval at different post-inoculation days (PID), if all values were below the detection limit datapoint is set at 0.00001. Number of included mink varies according to date of euthanasia, i.e., PID −2–14 (*n* = 24), PID 15–35 (*n* = 16), PID 36–70 (*n* = 8).

In the IP-inoculated group, AMDV was detected in serum from 1/4 mink at PID 5 in each of the groups of mink euthanized at 2 and 5 WPI, increasing to 3/4 positive mink at PID 12 in both groups. At PID 19 onwards, AMDV was detected in serum in both groups. In the group of mink euthanized at 10 WPI, all mink (*n* = 4) were viremic from PID 12 until euthanasia. AMDV was detected in all organs from all mink upon euthanasia after 2, 5, or 10 WPI. The level of virus was slightly lower in the organs of mink necropsied after 2 WPI and highest after 5 WPI. In the IP-inoculated group, the number of viremic animals and the level of virus in the positive animals developed earlier and faster than in the AE-inoculated mink (Table 2), but the level of virus in serum reached comparable levels. A summary of the infection dynamics of the AE and IP-inoculated mink, showing the development of the viral load in serum, liver, and lung, is presented in Figure 5.

**Figure 6:**
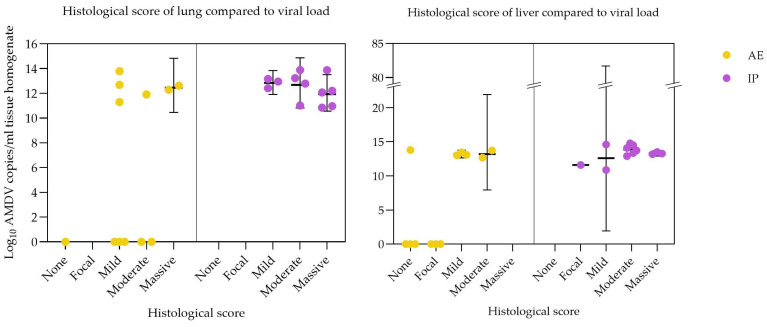
Scatter plots showing Log_10_ viral copies of Aleutian Mink Disease Virus (AMDV)/mL tissue homogenate in relation to the degree of liver and lung inflammation in all mink inoculated with AMDV via aerosol (●) and intraperitoneal (●) routes and necropsied at 2, 5, or 10 weeks post-inoculation. Inflammation, represented by infiltration of mononuclear cells, was scored histologically as: none (not present), focal (small focal infiltration), mild (few and small infiltrations), moderate (several small or few disseminated infiltrations), or massive (multifocal disseminated infiltrations). Presented as geometrical means with 95% confidence interval.

When comparing histology and PCR results for the lung and liver of all AE and IP AMDV-inoculated mink, a positive association between the viral load and the severity of histological changes was seen (Figure 6). Overall, no or only mild lesions were seen in organs with no or low viral loads, whereas increasing viral loads were associated with higher histological scores. This association was especially pronounced in the IP-inoculated mink, which generally had higher viral loads and exhibited more severe lesions in both liver and lung compared to the AE group. In the lungs of the AE-inoculated mink, there was greater variation, with some mink having high viral loads but only minor histological changes, while others had moderate lesions without detection of viral DNA.

No sentinels seroconverted before euthanasia, but virus was detected in organs and or serum of the four sentinels exposed to IP-inoculated mink during experimental weeks 3–6 (Table 5 and Table 6). AMDV was not detected in the sentinels exposed during weeks 1–2 and 7–10, in either organs or in serum. In the sentinels exposed during weeks 3–4, the virus was detected in the serum of 1/2 mink from PID 19, corresponding to 5 days after introduction to the IP-inoculated mink, until euthanasia. In the other sentinel, AMDV was detected from PID 40, corresponding to 26 days after exposure to IP-inoculated mink (Table 6). At necropsy, all examined organs were virus-positive in these two sentinels. In the 5–6-week exposure group, one mink was virus-positive in serum at PID 54, i.e., 26 days after introduction to the IP-inoculated mink. The other mink in this group remained serum negative. Among the AMDV-positive sentinels, the highest viral load in serum was detected in the 3–4-week exposed mink upon euthanasia, and AMDV was detected in all organs, with the highest viral load in the liver and spleen and the lowest in the brain. The two sentinels exposed during weeks 5–6 were AMDV-positive in organs, but only in 4/7 organs (AMDV not detected in serum) and 6/7 organs (AMDV detected in serum), respectively, with the highest viral load in the mesenteric lymph node and the lowest load in the brain and intestine. The viral loads in organs of these sentinels were substantially lower and showed greater variation between the two mink, compared to those of the sentinels from exposure weeks 3–4.

## 4. Discussion

In the present study, aiming to develop a disease model that reflects natural AMDV infection, we successfully established an aerosol (AE) inoculation model for AD in mink. The AE inoculation resulted in infection, defined by detection of viral DNA by PCR in organs and/or serum in six out of 12 inoculated mink. AMDV-specific antibodies were detectable from 4 WPI. The AE model was compared with the “classical” IP inoculation, which induced consistent AD in all 12 mink in the group; however, this method involves high viral doses administered directly into the abdominal cavity and does not reflect natural exposure. As expected, the IP-inoculated mink generally had higher viral loads and more pronounced liver lesions than those AE-inoculated. Similar to the IP model, the infection developed gradually over time with an increasing number of animals being affected at later time points, which may indicate that this is also the case when mink are naturally infected in the field. The number of viremic IP animals and the level of virus in these developed earlier and faster than in the AE-inoculated mink; however, at the end of the study, the level of virus in serum reached comparable levels. Furthermore, using timely replacements of sentinel mink, we observed that the risk of horizontal transmission of AMDV between mink was highest during the early phase of infection.

The observed pathology, both gross and histopathology, corresponded to that reported by others after experimental and natural infection with increasing incidence and severity of lesions related to duration of infection [5,22,23]. However, lung lesions are often reported as fewer and milder or absent compared to lesions in the liver and kidney [5,22]. This was not the case in our study, since mononuclear cell infiltrations were seen in the lungs in all experimental groups—negative controls, AMDV-inoculated mink, and sentinels. Lung lesions, some extensive, were also present in the lungs of mink where AMDV was not detected, including also the mock-inoculated negative control mink (AE-neg, IP-neg). As expected, the pulmonary inflammatory reaction was more pronounced in the AMDV-infected mink at the end of the study; otherwise there was no clear association between inoculation route or infection status. No signs of respiratory disease, like sneezing, coughing, dyspnea, or conjunctivitis, were observed during the experiment, and, therefore, it remains unclear what caused the observed pulmonary inflammation, but it is likely a non-specific response caused by environmental factors like dust, humidity, ventilation, and/or previous respiratory infections. Such unspecific pulmonary inflammation, not related to AD, has also been observed in other AMDV inoculation studies [23]. In a study by Jensen et al. (2016) [5], fewer lung lesions were observed among the AMDV-inoculated mink compared to our study, even though the experimental facility was the same. This may rely on the use of different Saeby AMDV strains with varying virulence or may be due to the non-specific “background” inflammation in the lungs of all experimental groups in this study. It is difficult to assess to which extent the observed lung changes were caused by the AMDV or mock inoculation, or this non-specific reaction. However, there was a slight tendency to see moderate and massive lung changes in the AMDV-infected mink. Thus, mononuclear cell infiltrations that can be induced by AD can also be seen in the lung of mink without AMDV infection, as confirmed by diagnostic submissions (authors’ personal experience) and previous studies [23,24]. Therefore, the lung is not particularly useful for histological diagnosis of AD in mink, an important consideration when sampling tissues for diagnostic pathology. This also applies to the intestine, which only scored “moderate” in the interobserver agreement analyses. However, immunohistochemistry could be performed to identify AMDV in tissue sections and should be used to differentiate between AMDV-specific and non-specific pulmonary inflammation in future studies. Contrary to our expectations, the aerosol-inoculated mink did not have a higher incidence or more severe pulmonary changes compared to the IP-inoculated mink, nor among the negative controls, nor the AMDV-inoculated mink. This indicates that the aerosol inoculation itself does not induce pronounced pulmonary inflammation. Surprisingly, there was also no clear difference in the amount of virus in the lungs of the AE and IP-inoculated mink, where we had expected to find higher AMDV levels in the AE-inoculated mink—at least early in the experiment. However, as expected, we confirmed that IP inoculation favors the liver, as the inflammation and viral load in the liver were slightly increased in the IP-inoculated animals compared with the AE-inoculated and the mink-to-mink infected sentinels. Another indication that IP favors the abdominal organs was that the IP-inoculated mink necropsied 5 and 10 WPI seemed to have slightly more virus in the abdominal organs (spleen, liver, intestine, mesenteric lymph node, and kidney) compared to the lung and brain. A possible mechanism is that the virus-containing inoculum is taken up by macrophages and lymphocytes [25,26], and then transported to the abdominal organs, where the spleen and liver, due to their size and high blood flow, are exposed to many of these virus-containing inflammatory cells. The virus distribution in the other organs was quite similar between the experimental groups. In general, the amount of AMDV was lowest in the brain or lung and highest in the mesenteric lymph node of the AMDV-positive mink (both AE and IP-inoculated, and sentinels). However, the number of animals in each experimental group was low; therefore, it was not possible to perform reliable statistical analyses. The low number of animals was also reflected in the observed variation in viral loads and histopathology of the organs, both within and between the groups.

All sentinels that were exposed to infection from the IP-inoculated mink during the experimental weeks 3–6 became infected; however, only the sentinels at risk during weeks 3–4 had virus in all organs, and also, the viral load of these organs was higher compared with that of the mink exposed during weeks 5–6. This indicates that the risk of AMDV transmission is highest during the early phase of the infection, with the greatest infection potential during weeks 3–4 after infection. Interestingly, both our study and a study by Jensen et al. (2014) [27] indicate that AMDV transmission, reflected as natural infection of sentinels, occurs around 3–4 weeks after infection. Jensen et al. (2014) [27] based this on the delayed seroconversion of the sentinels, which did not occur until 6–7 weeks after first exposure to AMDV-inoculated mink. The late seroconversion in naturally infected sentinels is consistent with our results, where none of the sentinels developed detectable antibodies by the time of euthanasia, 28 days after the first contact with IP-inoculated mink, despite being PCR-positive in serum and organs. Due to the small number of sentinels, these results can only be seen as tendencies and should be verified in a large-scale study. However, regarding AMDV biosecurity, it is important to know the peak transmission risk and, therefore, follow-up studies with more sentinels should be carried out to confirm these results. In practice, the delayed seroconversion implies that the virus has spread on the farm before antibodies can be detected in blood samples. Therefore, to reduce the risk of spread of AMDV in mink farms, it is important to establish efficient eradication strategies and not only stamp out AMDV antibody-positive animals, but also neighboring mink. Furthermore, for optimal early detection of AD, the AMDV status of mink should be assessed by PCR for viral detection in serum. The caveat of screening whole farms with PCR would be a higher cost compared to antibody screening, and access to a fast and reliable high-throughput AMDV PCR test. However, several AMDV strains with known differences in virulence exist; therefore, the course of infection depends on the specific strain, but also the viral dose and genotype of the host, all of which must be taken into consideration [10,12,13].

As in previous experiments [5,13], IP inoculation proved to be a reliable and effective model for inducing AMDV infection, since all IP-inoculated mink became infected, and virus was detected in all examined organs. We successfully established a model for AMDV AE inoculation of mink using a nebulizer chamber coupled to an anesthetic mask. Other studies have used aerosol inoculation for inducing AD in mink. Some of these studies used “aerosol inoculation boxes” [11,28], where the entire mink was exposed to the viral inoculum, causing virus contamination of the fur, and the virus will also be ingested by the mink during grooming. Another more recent study used intranasal inoculation, where the fluid inoculum was deposited directly into the nostrils [16], thus not expected to reach the lungs in the same way as fomites/aerosols are able to. When using a nebulizer chamber and an anesthetic mask covering only the mouth and nose, this has the advantage that the fur is not contaminated with the virus, thus providing a more controlled aerosol inoculation model. One challenge with the method was that the inhaled inoculum dose varied among the mink, as it relied on the respiratory rate of the anesthetized animals. Due to welfare concerns, the mink were only allowed to inhale the inoculum for a maximum of three minutes. However, this variation in dose was not reflected in the histopathology or PCR results. To our knowledge, this is the first time mink have been successfully infected with AMDV via AE inoculation using a nebulizer chamber and anesthetic mask. In the present study, after AE inoculation, the AMDV infection progressed gradually. Accordingly, at 2 WPI, none of the organs in the AE-inoculated mink contained detectable levels of AMDV, even though some of the AE-inoculated mink were viremic on day 12. However, since no animals were necropsied before 2 WPI, it cannot be ruled out that there was replication in the lung at earlier timepoints. Further studies are needed to clarify this. In contrast, after 5 and 10 WPI, virus was detected in the organs of 50% and 100% of the AE-inoculated mink, respectively, while all organs were already AMDV-positive in the IP mink necropsied at 2 WPI. The viral load in serum also developed gradually in the AE group. Thus, the first positive serum sample contained relatively fewer virus copies compared with the subsequent sampling, where the viral loads had increased substantially and were comparable to those of the IP-inoculated, who presented with these high viral loads in serum from the first detection. Thus, the pathogenesis of the AE-inoculated mink is expected to resemble the course of natural infection more closely compared with IP inoculation. However, the varying virulence of different AMDV strains and mink genotypes might affect the pathogenesis of AD; therefore, the AE inoculation model should be tested using other strains and genotypes. The sentinels represent the true natural mink-to-mink infection, and when compared with this, the AE inoculation resulted in a more homogeneous virus spread, with all examined organs being virus-positive at the end of the study, which was not the case in the infected sentinels.

## 5. Conclusions

In the present study, we succeeded in establishing an AMDV aerosol inoculation model for infection of mink using a nebulizer chamber and anesthesia mask. The aerosol model is considered to reflect the pathogenesis of natural infection better than the traditional IP inoculation. Transmission studies using sentinels indicated that the risk of passing on AMDV infection between mink is greatest early in the course of the infection, before antibody-positive animals can be found on the farm, which is crucial knowledge for combating AD.

## Figures and Tables

**Table 1:** Scoring of histological changes in organs from mink euthanized 2, 5, and 10 weeks after aerosol or intraperitoneal inoculation with mock (group AE-neg and IP-neg) or Aleutian Mink Disease Virus (AMDV) (group IP and AE). The reported scorings are averages of duplicate histological examinations. Total = number of mink with changes/number of mink in the group. Lesions in the lung, liver, small intestine, kidney, and brain were scored according to infiltration of mononuclear cells, as follows: focal (small focal infiltration); mild (few and small infiltrations); moderate (several small or few disseminated infiltrations) or massive (multifocal disseminated infiltrations).

Organ Score/ Group	2 Weeks Post-Inoculation				5 Weeks Post-Inoculation				10 Weeks Post-Inoculation			
	IP-Neg	AE-Neg	AE	IP	IP-Neg	AE-Neg	AE	IP	IP-Neg	AE-Neg	AE	IP
**Lung**												
Focal	1	-	-	-	-	-	-	-	1	-	-	-
Mild	1	-	3	-	-	-	1	2	1	-	2	1
Moderate	-	1	-	1	2	2	3	2	-	-	-	1
Massive	-	1	-	3	-	-	-	-	-	2	2	2
**Total**	**2/2**	**2/2**	**3/4**	**4/4**	**2/2**	**2/2**	**4/4**	**4/4**	**2/2**	**2/2**	**4/4**	**4/4**
**Liver**												
Focal	1	1	2	1	1	2	1	-	-	1	-	-
Mild	-	-	-	2	-	-	1	-	2	-	2	-
Moderate	-	-	-	1	-	-	-	4	-	-	2	1
Massive	-	-	-	-	-	-	-	-	-	-	-	3
**Total**	**1/2**	**1/2**	**2/4**	**4/4**	**1/2**	**2/2**	**2/4**	**4/4**	**2/2**	**1/2**	**4/4**	**4/4**
**Intestine**												
Focal	-	-	2	-	-	-	-	1	-	1	1	1
Mild	-	-	-	1	-	-	2	1	1	-	2	2
Moderate	-	-	-	-	-	-	-	-	-	-	-	-
Massive	-	-	-	-	-	-	-	-	-	-	-	-
**Total**	**0/2**	**0/2**	**2/4**	**1/4**	**0/2**	**0/2**	**2/4**	**2/4**	**1/2**	**1/2**	**3/4**	**3/4**
**Kidney**												
Focal	-	-	-	-	1	-	-	1	1	-	-	-
Mild	-	-	-	1	-	-	1	2	-	-	2	-
Moderate	-	-	-	-	-	-	-	1	-	-	-	-
Massive	-	-	-	-	-	-	-	-	-	-	2	4
**Total**	**0/2**	**0/2**	**0/4**	**1/4**	**1/2**	**0/2**	**1/4**	**4/4**	**1/2**	**0/2**	**4/4**	**4/4**
**Brain**												
Focal	-	1	-	1	-	-	-	-	-	1	1	-
Mild	-	-	-	1	-	-	-	1	-	-	1	2
Moderate	-	-	-	1	-	-	-	3	-	-	1	2
Massive	-	-	-	-	-	-	-	-	-	-	1	-
**Total**	**0/2**	**1/2**	**0/4**	**3/4**	**0/2**	**0/2**	**0/4**	**4/4**	**0/2**	**1/2**	**4/4**	**4/4**

**Table 2:** Serum PCR results from mink euthanized 2, 5, and 10 weeks after aerosol or intraperitoneal inoculation with mock (group AE-neg and IP-neg) or Aleutian Mink Disease Virus (AMDV) (group IP and AE). Number of positive animals out of the number of tested animals, and average of AMDV copies pr. ml serum for positive animals. WPI, euthanasia at week post-inoculation. Nd, not determined; -, not relevant.

		Post-Inoculation Day											
Group	WPI	−2	5	12	19	26	33	35	40	47	54	61	70
AE-neg	2	0/2	Nd	0/2	-	-	-	-	-	-	-	-	-
	5	0/2	0/2	0/2	0/2	0/2	0/2	0/2	-	-	-	-	-
	10	Nd	0/2	0/2	0/2	0/2	0/2	Nd	0/2	0/2	0/2	0/2	0/2
IP-neg	2	0/2	Nd	0/2	-	-	-	-	-	-	-	-	-
	5	0/2	Nd	0/2	0/2	0/2	0/2	0/2	-	-	-	-	-
	10	0/2	Nd	0/2	0/2	0/2	0/2	Nd	0/2	0/2	0/2	0/2	0/2
AE	2	0/4	0/4	0/4	-	-	-	-	-	-	-	-	-
	5	0/4	0/4	**2/4**	**1/4**	**1/4**	**2/4**	**2/4**	-	-	-	-	-
		0	0	7.56 × 10^3^	1.83 × 10^11^	9.19 × 10^10^	8.71 × 10^10^	1.05 × 10^13^	-	-	-	-	-
	10	0/4	0/4	**2/4**	**3/4**	**3/4**	**3/4**	Nd	**4/4**	**4/4**	**4/4**	**4/4**	(4/4) *
		0	0	2.26 × 10^5^	1.66 × 10^12^	2.82 × 10^13^	1.22 × 10^11^	-	1.39 × 10^13^	6.92 × 10^10^	1.01 × 10^13^	5.16 × 10^13^	5.12 × 10^13^
IP	2	0/4	**1/4**	**3/4**	-	-	-	-	-	-	-	-	-
		0	2.02 × 10^12^	7.02 × 10^6^	-	-	-	-	-	-	-	-	-
	5	0/4	**1/4**	**3/4**	**4/4**	**4/4**	**4/4**	**4/4**	-	-	-	-	-
		0	6.47 × 10^10^	1.54 × 10^14^	1.22 × 10^12^	5.41 × 10^13^	6.92 × 10^10^	2.59 × 10^13^	-	-	-	-	-
	10	0/4	0/4	**4/4**	**4/4**	**4/4**	**4/4**	Nd	**4/4**	**4/4**	**4/4**	**4/4**	**4/4**
		0	0	1.67 × 10^12^	4.57 × 10^11^	5.33 × 10^13^	2.42 × 10^10^	-	2.10 × 10^12^	2.26 × 10^10^	6.64 × 10^12^	1.02 × 10^14^	5.72 × 10^9^

The proportion of positive mink is marked in bold. * Two AE-inoculated mink were euthanized due to welfare reasons and tested on PID 68; the results are reported together with the two remaining mink euthanized on PID 70.

**Table 3:** Organ PCR results from mink euthanized 2, 5, and 10 weeks after aerosol or intraperitoneal inoculation with mock (group AE-neg and IP-neg) or Aleutian Mink Disease Virus (AMDV) (group IP and AE). Number of positive animals out of the number of animals in the group, average of AMDV copies pr. ml tissue homogenate for positive animals. WPI, euthanasia at week post-inoculation.

		PCR-Positive Organs						
Group	WPI	Spleen	Liver	Lung	Kidney	Intestine	Lymph Node †	Brain
	2	0/2	0/2	0/2	0/2	0/2	0/2	0/2
AE-neg	5	0/2	0/2	0/2	0/2	0/2	0/2	0/2
	10	0/2	0/2	0/2	0/2	0/2	0/2	0/2
	2	0/2	0/2	0/2	0/2	0/2	0/2	0/2
IP-neg	5	0/2	0/2	0/2	0/2	0/2	0/2	0/2
	10	0/2	0/2	0/2	0/2	0/2	0/2	0/2
	2	0/2	0/2	0/2	0/2	0/2	0/2	0/2
AE	5	**2/4**	**2/4**	**2/4**	**2/4**	**2/4**	**2/4**	**2/4**
		3.3 × 10^13^	3.7 × 10^13^	5.1 × 10^11^	3.6 × 10^12^	5.5 × 10^13^	1.5 × 10^15^	4.4 × 10^12^
	10	**4/4**	**4/4**	**4/4**	**4/4**	**4/4**	**4/4**	**4/4**
		6.6 × 10^13^	2.4 × 10^13^	1.8 × 10^13^	3.2 × 10^13^	4.1 × 10^13^	7.0 × 10^15^	6.6 × 10^12^
	2	3 */4	**4/4**	**4/4**	**4/4**	**4/4**	**4/4**	**4/4**
		2.0 × 10^12^	1.0 × 10^14^	1.9 × 10^13^	2.2 × 10^12^	1.9 × 10^12^	8.3 × 10^13^	1.1 × 10^11^
IP	5	**4/4**	**4/4**	**4/4**	**4/4**	**4/4**	**4/4**	**4/4**
		7.0 × 10^14^	2.4 × 10^14^	2.7 × 10^13^	1.6 × 10^14^	2.3 × 10^14^	2.8 × 10^15^	1.3 × 10^13^
	10	**4/4**	**4/4**	**4/4**	**4/4**	**4/4**	**4/4**	**4/4**
		4.1 × 10^13^	4.7 × 10^13^	5.6 × 10^12^	3.2 × 10^13^	4.4 × 10^13^	3.1 × 10^14^	2.2 × 10^12^

The proportion of positive mink is marked in bold. † Mesenteric lymph node. * Spleen from only 3 mink tested.

**Table 4:** Aleutian Mink Disease Virus (AMDV) antibodies in the groups of aerosol (AE) or intraperitoneal (IP) mock-inoculated mink (AE-neg, IP-neg), and AE or IP AMDV-inoculated (number of positive blood samples/number of mink in the group) relative to time in the experiment. Two mink from group IP-neg and AE-neg, respectively, and 4 mink from group AE and IP, respectively, were euthanized 2, 5, and 10 weeks after inoculation. None of the sentinels seroconverted.

	Post-Inoculation Day (Weeks Post-Inoculation)										
Group	−2 (0)	5 (1)	12 (2)	19 (3)	26 (4)	33 (5)	40 (6)	47 (7)	54 (8)	61 (9)	68 (10)
AE-neg, IP-neg	0/12	0/12	0/12	0/8	0/8	0/8	0/4	0/4	0/4	0/4	0/4
AMDV AE	0/12	0/12	0/12	0/8	**4/8**	**4/8**	**3/4**	**3/4**	**4/4**	**4/4**	**4/4**
AMDV IP	0/12	0/12	0/12	**2/8**	**8/8**	**8/8**	**4/4**	**4/4**	**4/4**	**4/4**	**4/4**

The proportion of positive mink is marked in bold.

**Table 5:** Aleutian Mink Disease Virus (AMDV) PCR results (Cq-values) of organs from individual sentinel mink exposed during two-week periods, to mink intraperitoneally inoculated (IP) with AMDV. 0, AMDV not detected. Weeks post-inoculation (WPI) of IP mink.

		PCR-Positive Organs (Cq-Values)						
Mink No.	WPI Exposed to IP Mink	Spleen	Liver	Lung	Kidney	Intestine	Lymph Node *	Brain
1	1–2	0	0	0	0	0	0	0
2		0	0	0	0	0	0	0
3	3–4	**12.47**	**15.06**	**14.75**	**22.95**	**15.77**	**12.48**	**25.09**
4		**13.23**	**11.01**	**16.40**	**22.58**	**17.68**	**14.18**	**26.01**
5	5–6	**31.32**	**34.08**	**34.48**	0	0	**25.57**	0
6		**26.97**	**24.67**	**26.92**	0	**31.78**	**15.96**	**31.37**
7	7–8	0	0	0	0	0	0	0
8		0	0	0	0	0	0	0
9	9–10	0	0	0	0	0	0	0
10		0	0	0	0	0	0	0

Positive results are marked in bold. * Mesenteric lymph node.

**Table 6:** Aleutian Mink Disease Virus (AMDV) PCR results in serum from individual sentinel mink exposed during two-week periods to mink intraperitoneally inoculated (IP) with AMDV. 0, AMDV not detected by PCR; for positive serum samples, the copy numbers of AMDV/mL serum are given. Time interval at risk (exposed to IP-inoculated mink) is indicated by light purple marking. Two weeks period after exposure ended is indicated by light grey marking; euthanasia was on the last day of this period. Notice that the timeline is not to scale. Nd, not determined; -, not relevant.

	WPI Exposed to IP Mink	Post Inoculation Day																
Mink No.		−2	0	5	12	14	19	26	28	33	40	42	47	54	56	61	70	84
1	1–2	0	-	0	0	-	0	0	0									
2		0	-	0	0	-	0	0	0									
3	3–4				Nd	-	1.90 × 10^6^	-	-	2.00 × 10^8^	6.27 × 10^12^	2.33 × 10^11^						
4					Nd	-	0	-	-	0	2.16 × 10^12^	2.59 × 10^10^						
5	5–6							0	-	0	0	-	0	0	0			
6								0	-	0	0	-	0	4.72 × 10^4^	5.08 × 10^8^			
7	7–8										0	-	0	0	-	0	0	
8											0	-	0	0	-	0	0	
9	9–10													0	-	0	0	0
10														0	-	0	0	0

## Data Availability

The datasets used for this study are available from the corresponding author upon reasonable request.

## Supplementary Materials

The following supporting information can be downloaded at: https://www.mdpi.com/article/10.3390/pathogens15050494/s1, Table S1: AMDV PCR result (copies/ml tissues homogenate or serum) in organs and serum, and AMDV antibody (Ab) results, upon necropsy.; Table S2: Antibody (Ab) and Serum qPCR result (copies/ml serum) for individual mink and average qPCR results of positive mink pr. group.

## Abbreviations

The following abbreviations are used in this manuscript:

AE	Aerosol
AE group	Mink aerosol-inoculated with Aleutian Mink Disease Virus
AE-neg group	Negative control mink, aerosol-inoculated with mock
AD	Aleutian disease
AMDV	Aleutian Mink Disease Virus
DBS cards	Dried blood spot cards
DTU-Vet	The National Veterinary Institute, Technical University of Denmark
IM	Intramuscular
IP	Intraperitoneal
IP group	Mink inoculated intraperitoneally with Aleutian Mink Disease Virus
IP-neg group	Negative control mink, inoculated intraperitoneally with mock
PID	Post-inoculation day
qPCR	Quantitative real-time PCR
WPI	Weeks post-inoculation
