# Supplementary material for: Pathogenesis of Aleutian Mink Disease Virus Infection—Comparison of Natural Transmission with Experimental Aerosol and Intraperitoneal Inoculation
Source: Pathogens. 2026 May 3;15(5):494. doi: 10.3390/pathogens15050494 (PMC13209166; doi:10.3390/pathogens15050494)
Supplement: Supplementary file 1 [file pathogens-15-00494-s001.zip › Supplementary Table 1_organs_April 2026.pdf]

Supplementary table S1. AMDV PCR result (copies/ml tissues homogenate or serum) in organs and serum, and AMDV antibody (Ab) results, upon necropsy. neg, Ab not detected; IP, AMDV intraperitoneal inoculation; AE, AMDV aerosol inoculation; 0, AMDV not detected; Nd, not determined; Ab, AMDV specific antibodies.

Mink No.	Group	Euthanasia week	Spleen	Liver	Lung	Kidney	Brain	Duodenum	Mes. Lymph node	Serum, virus	Serum, Ab
101-102	IP-neg	2	0	Nd	Nd	Nd	Nd	Nd	Nd	0	neg
103-104	IP-neg	5	0	Nd	Nd	Nd	Nd	Nd	Nd	0	neg
105-106	IP-neg	10	0	Nd	Nd	Nd	Nd	Nd	Nd	0	neg
107-108	AE-neg	2	0	Nd	Nd	Nd	Nd	Nd	Nd	0	neg
109-110	AE-neg	5	0	Nd	Nd	Nd	Nd	Nd	Nd	0	neg
111-112	AE-neg	10	0	Nd	Nd	Nd	Nd	Nd	Nd	0	neg
113-116	AE	2	0	0	0	0	0	0	0	0	neg
117	AE	5	0	0	0	0	0	0	0	0	Nd
118	AE	5	1.72E+10	6.23E+13	2.02E+11	6.13E+10	1.06E+10	2.74E+12	3.83E+14	3.30E+11	Nd
119	AE	5	6.61E+13	1.21E+13	8.14E+11	7.19E+12	8.82E+12	1.07E+14	2.58E+15	2.06E+13	Nd
120	AE	5	0	0	0	0	0	0	0	0	Nd
117-120	AE	Average	**3.30E+13**	**3.72E+13**	**5.08E+11**	**3.62E+12**	**4.42E+12**	**5.47E+13**	**1.48E+15**	1.05E+13	
121	AE	10	4.77E+12	4.77E+12	1.93E+12	3.37E+12	3.01E+12	1.85E+13	4.40E+14	Nd	Nd
122	AE	10	1.43E+14	5.40E+13	6.13E+13	1.20E+14	3.67E+12	9.33E+13	4.63E+14	1.01E+14	Nd
123	AE	10	9.40E+13	2.77E+13	4.23E+12	4.10E+11	2.33E+11	7.17E+12	7.30E+14	Nd	Nd
124	AE	10	2.32E+13	1.02E+13	4.87E+12	6.97E+12	1.96E+13	4.47E+13	1.16E+15	1.57E+12	Nd
121-124	AE	Average	**6.63E+13**	**2.42E+13**	**1.81E+13**	**3.26E+13**	**6.63E+12**	**4.09E+13**	**6.99E+14**	5.13E+13	
125	IP	2	4.99E+12	7.73E+12	7.12E+10	8.76E+12	3.75E+11	7.26E+12	2.20E+14	9.00E+14	Nd
126	IP	2	Nd	7.43E+10	9.22E+10	3.65E+07	3.09E+07	7.00E+09	2.25E+12	4.38E+14	Nd
127	IP	2	1.13E+12	3.90E+14	7.37E+13	1.01E+11	4.77E+10	2.30E+11	9.73E+13	2.45E+14	Nd
128	IP	2	1.14E+10	4.03E+11	1.04E+11	1.02E+10	8.27E+08	1.40E+11	1.15E+13	1.43E+11	Nd
125-128	IP	Average	**2.04E+12**	**9.95E+13**	**1.85E+13**	**2.22E+12**	**1.06E+11**	**1.91E+12**	**8.27E+13**	3.96E+14	
129	IP	5	3.77E+13	2.17E+13	6.30E+12	1.78E+13	8.80E+11	1.25E+13	6.02E+14	7.01E+13	Nd
130	IP	5	2.31E+14	5.20E+13	9.07E+12	3.20E+13	5.39E+12	1.97E+13	1.25E+15	3.14E+13	Nd
131	IP	5	2.09E+15	5.83E+14	7.67E+13	5.73E+14	3.63E+13	7.23E+14	5.37E+15	1.68E+12	Nd
132	IP	5	4.40E+14	3.12E+14	1.46E+13	1.97E+13	8.00E+12	1.60E+14	3.90E+15	5.00E+11	Nd
129-132	IP	Average	**6.99E+14**	**2.42E+14**	**2.66E+13**	**1.61E+14**	**1.26E+13**	**2.29E+14**	**2.78E+15**	2.59E+13	

133	IP	10	1.53E+13	1.79E+13	1.69E+13	2.83E+13	3.43E+12	5.77E+13	1.26E+14	1.02E+10	Nd
134	IP	10	2.19E+13	3.13E+13	1.20E+12	1.97E+13	1.81E+12	8.00E+13	8.17E+13	8.30E+09	Nd
135	IP	10	1.12E+14	1.22E+14	1.61E+12	6.10E+13	1.06E+12	1.23E+13	7.93E+14	4.19E+09	Nd
136	IP	10	1.40E+13	1.52E+13	2.61E+12	2.07E+13	2.43E+12	2.60E+13	2.36E+14	2.30E+08	Nd
133-136	IP	Average	**4.08E+13**	**4.67E+13**	**5.58E+12**	**3.24E+13**	**2.18E+12**	**4.40E+13**	**3.09E+14**	5.72E+09	
